# Linking the foraging behavior of three bee species to pollen dispersal and gene flow

**DOI:** 10.1371/journal.pone.0212561

**Published:** 2019-02-26

**Authors:** Johanne Brunet, Yang Zhao, Murray K. Clayton

**Affiliations:** 1 USDA-ARS, VCRU, Department of Entomology, University of Wisconsin, Madison, Wisconsin, United States of America; 2 Department of Statistics, University of Wisconsin, Madison, Wisconsin, United States of America; Universidade Federal de Uberlândia, BRAZIL

## Abstract

Foraging behaviors that impact gene flow can guide the design of pollinator strategies to mitigate gene flow. Reduced gene flow is expected to minimize the impact of genetically engineered (GE) crops on feral and natural populations and to facilitate the coexistence of different agricultural markets. The goal of this study is to link foraging behavior to gene flow and identify behaviors that can help predict gene flow for different bee species. To reach this goal, we first examined and compared the foraging behaviors of three distinct bee species, the European honey bee, *Apis mellifera L*., the common eastern bumble bee, *Bombus impatiens Cr*., and the alfalfa leafcutting bee, *Megachile rotundata F*., foraging on *Medicago sativa* flowers. Each foraging behavior investigated differed among bee species. Both social bees exhibited directionality of movement and had similar residence, in contrast to the random movement and shorter residence of the solitary bee. Tripping rate and net distance traveled differed among the three bee species. We ranked each behavior among bee species and used the relative ranking as gene flow predictor before testing the predictions against empirical gene flow data. Tripping rate and net distance traveled, but not residence, predicted relative gene dispersal among bee species. Linking specific behaviors to gene flow provides mechanisms to explain differences in gene flow among bee species and guides the development of management practices to reduce gene flow. Although developed in one system, the approach developed here can be generalized to different plant/pollinator systems.

## Introduction

Insects are important pollinators both in natural habitats and in agricultural systems. Globally, over 87% of flowering plants and approximately 35% of crops benefit from animal pollination [1–2]. The vast majority of fruits and vegetables, some forage crops (alfalfa and clovers) and oil-producing crops (canola), require insects for pollination and subsequent seed production [3]. Insect pollinators transfer pollen as they move from flower to flower, and when a seed is set, they move the genes transmitted via the pollen. The movement of genes by insect pollinators can lead to the escape and subsequent spread of genetically engineered traits and create adventitious presence or unwanted gene flow [4]. Concern about unwanted gene flow has increased as a result of the greater acreages planted to genetically engineered (GE) crops and the larger number of GE traits. Unwanted gene flow affects not only seed-production fields destined to markets with distinct levels of tolerance for GE traits [5] such as the conventional, organic and export markets, but also feral and wild populations [6–7]. Despite the forthcoming increase in the number of insect-pollinated GE crops, our knowledge of the factors that influence the movement of genes via pollen by insect pollinators remains limited. A better understanding of how pollinator foraging behavior is linked to gene flow would improve predictions of gene flow risk for insect-pollinated plants. It would also guide the development of pollinator management strategies to reduce unwanted gene flow which, in turn, would facilitate the coexistence of different markets and limit the potential for introgression of transgenes into wild or feral populations [6].

Various pollinator foraging behaviors, including residence, distances traveled and tripping rate, are expected to affect gene flow. Residence represents the total number of flowers visited in a patch by a pollinator during a foraging bout while tripping rate is the proportion of flowers visited by a pollinator whose stigmas and anthers are released from the keel. Residence has been proposed as being inversely proportional to gene flow [8–9]. When pollinators move from a GE field to a conventional field of a crop, they are carrying GE pollen on their bodies. It is only the GE pollen on the areas of the pollinator’s body that come into contact with the flowers' stigmas that gets deposited on flowers visited in succession by the pollinator. The pollen stored in the pollen sacs is not involved in the pollination process and is brought back to the hive to feed the young [10–11]. The more flowers a pollinator visits in succession during a foraging bout, the greater the chance that most of the GE pollen be deposited in the first field visited and not be moved to the next field [9]. Therefore, high residence limits gene flow and residence is expected to be inversely proportional to gene flow [8].

Besides residence, the distances traveled by pollinators can affect gene flow. Pollinators pick up pollen from anthers and deposit pollen on stigmas as they move between successive flowers, inflorescences, and plants. The distance traveled by pollen will be generally shorter than the distance traveled by the pollinators [12]. Previous work by Levey et al. [13–14] demonstrated how distances and directions traveled by birds moving from perch to perch at a local scale could be used to model and predict gene flow patterns of the seeds they ingest at a landscape level. For pollinators, the net distance traveled will be affected by how they move among successively visited flowers, inflorescences and plants. Some pollinator species move randomly among flowers, inflorescences and plants, while others tend to move in the same direction within a foraging bout, a process known as directionality of movement [15]. Pollinators that exhibit directionality of movement will tend to move greater net distances relative to pollinators with random movements [16]. Pollinators that move greater net distances are expected to move pollen and genes further. In addition to the patterns of pollinator movement among flowers, the preference of pollinators for specific directions can influence the pattern of pollen dispersal and gene flow. Such preferences could result in biased pollen dispersal and gene flow over the landscape. To date, a pattern of non-uniform gene flow, where gene flow prevails in some directions, has not been examined in insect-pollinated plants. It has, however, been identified in some wind-pollinated plant species, where gene flow is biased in the direction of prevailing winds [17].

Pollen dispersal and gene flow can also be affected by the tripping rate or the proportion of visited flowers whose stigmas and anthers are exposed by a pollinator [12]. In certain plant species, including species in the legume family, bees must depress the keel petals at the base of the flower in order to release the anthers and style in a process called tripping. Tripping is necessary before pollination and subsequent seed set can occur. To illustrate the impact of tripping rate on pollen dispersal and gene flow, imagine a pollinator that carries GE pollen on its body and is moving into a field of conventional flowers and tripping a low proportion of the flowers it visits. Because pollen is not deposited on stigmas or removed from anthers of non-tripped flowers, more GE pollen remains on the pollinator's body and is available for pollinating flowers in the next field visited, relative to a pollinator that trips all the flowers visited in the first field. Therefore, GE pollen will move greater distances overall when a lower percentage of visited flowers are tripped by a pollinator. Lower tripping rate of flowers is therefore expected to increase pollen dispersal distances [12] and because tripping rate influences seed set [18], lower tripping rate is expected to increase gene dispersal distances.

In this study, we first examined and contrasted the behavior of three bee species, two social bees, the honey bee, *Apis mellifera*, and the common eastern bumble bee, *Bombus impatiens*, and a solitary bee, the alfalfa leafcutting bee, *Megachile rotundata*, foraging on *Medicago sativa* flowers. We focused on behaviors likely to affect gene flow. We also examined whether each bee species exhibited directionality of movement within foraging bouts and whether it displayed an overall preference for some directions. *Medicago sativa* is an open-pollinated plant with high levels of phenotypic and genetic variation and it is visited by different bee species. Two genetically engineered (GE) traits are currently commercially available, the Roundup Ready (RR) trait, which provides resistance to glyphosate, and a low-lignin content alfalfa which increases the digestibility of *M*. *sativa* to cows. The risk of unwanted gene flow and escape of GE genes is therefore a great concern for this plant species and RR genes have been detected in various feral populations [7]. Foraging behaviors and gene flow distances can vary among pollinator species [19–23] and some foraging behaviors may help predict differences in gene flow for distinct pollinator species. To link foraging behavior to gene flow, we ranked each foraging behavior among bee species, used the ranking to predict relative gene flow and tested predictions against empirical gene flow data. Differences in foraging behaviors provided mechanisms to explain observed differences in gene flow among bee species and pinpointed management strategies to mitigate gene flow. Given the current increase in GE crops over the agricultural landscape and the deployment of new gene editing technologies, the approach developed here can be expanded to benefit other insect-pollinated crops.

## Materials and methods

### Plant species and pollinators

*Medicago sativa* L. (Fabaceae), also known as alfalfa or lucerne, is an open-pollinated outcrossed perennial legume which relies on insects for pollination. Flowers are clustered into racemes and a plant can bear many stems with many racemes per stem and flowers per raceme [18]. Individual flowers can remain open for a week when not pollinated and multi-seeded pods take about six weeks to mature following pollination. Peak bloom for *M*. *sativa* occurs in July in south central Wisconsin where a *M*. *sativa* field tends to flower over a 4–6 week period.

Honey bees, *Apis mellifera* L., and alfalfa leafcutting bees, *Megachile rotundata* F., are used as managed pollinators in alfalfa seed-production fields. Honey bee is the dominant managed pollinator in California (CA) whereas the alfalfa leafcutting bee dominates in the Pacific Northwest but is becoming more important in CA. Several native pollinators, including bumble bees and some solitary bees, visit and pollinate *M*. *sativa* flowers [24, 23].

Bees forage for both pollen and nectar on *M*. *sativa* flowers. Bees trip alfalfa flowers permitting a simultaneous receipt of pollen on the stigma and deposition of pollen on the pollinator's body. Once a flower is tripped, the stigma and anthers remain pressed against the upper part of the flower.

### Experimental set up

Five 11 m × 11 m patches of *M*. *sativa* were set up in an east-west linear arrangement at the West Madison Agricultural Research Station (WMARS). This land belongs to and is rented from the University of Wisconsin, Madison. Permits are not required for University employees or affiliates to work at the WMARS facilities. This study did not involve endangered or protected species. Within each patch, 169 individual seedlings started in the greenhouse were transplanted 90 cm apart. Each summer, one honey bee hive was set up about 100 m from the patches; a bumble bee hive was placed in the center of the southern edge of the patches; and two boxes containing a 60 ×30 ×7.6 cm bee board, to allow leafcutting bees to nest, were set up 1/3 and 2/3 of the distance along the northern edge of the patches (facing southwest). A half-gallon of leafcutting bees was released at periodic intervals throughout the flowering season. The honey bee hive was located as described because honey bees would otherwise rarely visit the patches (J Brunet, pers. obs.). The same honey bee hive was used both years and consisted of two deeps and one honey super. The honey bee hive housed around 20,000 bees. Each bumble bee hive was purchased as a 75 workers hive from Koppert Biological Systems, Howell, MI, USA and placed in the field one to two weeks prior to the beginning of data collection to let the bees accommodate to their new environment. The bumble bee hive was placed in a wooden shelter set up approximately 0.5 meter off the ground.

Bee observations took place over two consecutive summers. Observers were trained to spot and follow the bees in the patches prior to the beginning of data collection. Bee observations were made throughout the flowering period of alfalfa and at the times of day when the bees were active. Observers went to the field to observe bees on almost all non-rainy days during the flowering period of *M*. *sativa*. Mornings tended to have greater bee activity, but the windows of time for bee activity varied somewhat by bee species and with the weather and time of year. Even though bees were brought to the field, pollinator abundance remained low in the patches such that individual bees could be followed and little interference was observed between bee species.

### Distance and direction traveled

When a pollinator was spotted in a patch, the bee species was noted and the pollinator was followed by three observers until it left the patch or was lost to the observers. Each raceme visited in succession by that pollinator was marked with a clip where each clip number indicated the order in which a raceme was visited within a foraging bout. After a bee had left the patch, the observers retraced each numbered clip and measured the distance (cm) and direction traveled by that bee between each pair of consecutively visited racemes. The direction traveled between each pair of racemes visited in succession was scored using the closest cardinal or inter-cardinal direction (N, NE, E, SE, S, SW, W or NW). A transparency marked with the eight directions and 22.5 degree intervals facilitated scoring of directions in the field.

From these observations, we obtained the distance and direction traveled between consecutive racemes and we used these distance and direction data to calculate the net distance traveled by a bee during a visit to a patch. The net distance traveled represented the difference between where a bee observation started and the position of the bee before it left the patch. We examined the frequency distribution of net distances traveled for each bee species. Finally, we computed the average number of clips (distances between two consecutive racemes) in a foraging bout for the three bee species.

### Residence

In a separate set of pollinator observations that took place throughout the alfalfa flowering period and at times of the day when bees were active, we obtained data on foraging bout duration and residence. Residence represents the total number of flowers visited in a patch during a complete foraging bout. A foraging bout started when a pollinator entered the patch and ended when it left the patch. We obtained residence data for bumble bees and honey bees; leafcutting bees proved too difficult to spot entering a patch. Residence and foraging bout duration were obtained only from complete foraging bouts.

### Tripping rate

In a third set of pollinator observations, we determined the tripping rate or the proportion of the flowers visited on a raceme that was tripped by each bee species. These data were collected throughout the alfalfa flowering period at different times throughout the day. When a pollinator approached a raceme, we noted the bee species and recorded the number of flowers visited and the number of flowers tripped on the raceme during the bee's visit. Previously tripped flowers on the raceme were not counted as tripped flowers. The detection of tripped flowers was facilitated by the fact that *M*. *sativa* flowers do not close again following tripping.

### Statistical analyses

#### Distances traveled

We examined the impact of bee species, year, and their interaction on the distances traveled between consecutive racemes and the net distances traveled during a foraging bout. For the distance traveled between consecutive racemes we used mixed linear models (Proc Mixed [25], SAS 9.4) where foraging bout was a random factor and bee species and year and their interaction were fixed effects. Distances were log transformed prior to analyses. For net distance traveled, we used a linear model with bee species and year and its interaction. Net distances were square root transformed prior to analyses. When a main factor was statistically significant and had more than two levels, we used protected (Fisher) multiple comparison t-tests to determine differences among pairs while controlling the overall (family-wise) error rate.

#### Directionality of movement within foraging bouts

Serial angular correlations were used to determine whether the directions of successive flight segments were correlated within foraging bouts, indicating directional persistence. A positive correlation indicates that, once a given forager starts moving in one direction, it tends to continue moving in that same direction throughout the foraging bout. While a statistic named W has been developed to measure similarity between successive directions within a single foraging bout [26–27], no such statistic is available to test directionality over a number of foraging bouts. We therefore modified the calculation of the W statistic [26] to apply to many foraging bouts in order to obtain an overall test of directionality of movement within foraging bouts. We created a test statistic, WS, which was the average of the individual W values. We applied a randomization test to evaluate the statistical significance of the empirically observed WS value. A randomization process to obtain one WS value included averaging the W values over all foraging bouts in the data set. Each W value was obtained by randomly shuffling the observed directional data in a foraging bout, which corresponded to a lack of serial angular correlation. This randomization process was performed 1000 times to generate the distribution of WS expected in the absence of serial angular correlation i.e. under the null hypothesis. The WS value calculated from the observed data was then compared to the WS distribution generated assuming no serial angular correlation. If the WS value of the observed data lied at the extreme of the WS distribution, indicating a small p value (P < 0.05), then we could reject the null hypothesis that no serial correlation existed within a foraging bout. A one-sided test was used since we were interested in positive correlations. Six separate tests were performed for serial angular correlation of directionality: one for bumble bees, one for leafcutting bees and one for honey bees in 2012 and again in 2013. For these tests we used all foraging bouts with more than two directions, i.e. foraging bouts with more than three racemes visited in succession. The directions were transformed into degrees prior to these analyses by setting the east position to 0° and north to 90°.

#### Residence and tripping rate

Linear mixed models (Proc Mixed [25]) were used to examine the impact of bee species on residence, i.e. the total number of flowers visited during a foraging bout in a patch, and on foraging bout duration. Each dependent variable was log transformed prior to analyses to meet the assumptions of the model. Because complete foraging bout data were not available for each bee species each year, year was not included in the model for residence or foraging bout duration. There was no random effect in this model; each data point summarized a foraging bout.

The impact of bee species, year and their interaction on the tripping rate was examined using a generalized linear model (Proc Glimmix [25]) with a binomial distribution and a logit link function. For the binomial analyses, the data were entered as number of flowers tripped and number of flowers visited per raceme.

#### Overall preference for directions

A uniformity test was used to examine preference of some overall directions by a bee species. Directions were first transformed into degrees with east representing 0° and north 90°. For each bee species each year we calculated the average direction traveled for each foraging bout. Mean directions were used to eliminate the potential effect of angular serial correlation within foraging bouts. The null hypothesis was a uniform probability of choosing a direction over all potential directions. Only foraging bouts with five or more racemes visited in succession were used because they provided a more continuous distribution of directions. The Watson test for Uniformity was selected because it has the greatest power among several uniformity tests including the Rayleigh test, Rao’s spacing test and the von Mises distribution test [27].

## Results

### Distances traveled

The average distance traveled between consecutive racemes varied significantly among bee species (F_2, 1875_ = 4.30, P = 0.014), but not between years (F_1, 1875_ = 3.10, P = 0.079) and there was no interaction between the effects of bee species and year on the distance traveled between consecutive racemes (F_2, 1875_ = 1.83. P = 0.16). The average distance traveled between consecutive racemes varied among foraging bouts (random effect estimate = 0.065, Z = 2.28; P = 0.011). Bumble bees and leafcutting bees traveled similar distances between consecutive racemes (BB: 21.12 cm (19.1, 23.3) and LCB: 20.70 cm (17.4, 24.7) (t_1, 1875_ = 0.13, P = 0.90) but both bee species traveled significantly greater distances relative to honey bees (HB: 15.80 cm (13.5, 18.5) (BB-HB: t_1, 1875_ = 2.86, P = 0.004 and HB-LCB: t_1, 1875_ = 2.14, P = 0.033). The distance data represent the back transformed least square means of the log distances and the corresponding 95% confidence intervals. These values were used because the mean of the raw data was affected by the long tail of the distribution before transformation. These analyses used 2183 distances collected over 308 foraging bouts.

Bee species varied significantly in the net distance traveled during a foraging bout (F_2, 302_ = 16.66, P < 0.001) with bumble bees traveling greater distances (mean +/- se) (BB: 213.7 +/- 14.6 cm) than honeybees (HB: 148.3 +/- 21.8 cm) (BB-HB: t_1, 302_ = 2.43, P = 0.016) and both bumble bees and honey bees travelling longer net distances relative to leafcutting bees (LCB: 72.2 +/- 19.1 cm) (t_1, 302_ = 5.75, P < 0.0001 for BB-LCB and t_1,302_ = 2.56, P = 0.011 for HB-LCB) (Table 1). We observed no significant differences in net distance traveled between years (F_1, 302_ = 0.00, P = 0.98) (2012 = 151.3 +/- 15.0 cm and 2013 = 138.1 +/- 15.6 cm) and no statistically significant interaction between bee species and year (F_2, 302_ = 1.19, P = 0.31). The frequency distribution of net distances traveled by each bee species was leptokurtic (S1 Fig). Honey bees had an average of (mean +/ SE) 12.4 +/- 2.8 clips per foraging bout relative to 10.7 +/- 1.5 for bumble bees and 4.2 +/- 0.49 for leafcutting bees.

**Table 1:** Relative ranking (1 is highest) or presence/absence of the different foraging behaviors for the three bee species.

Trait/Bee type	Honey bee	Bumble bee	Leafcutting bee
Distance traveled between racemes	3	1	1
*Net distance traveled and gene flow*	*2*	*1*	*3*
*prediction*			
Residence	1	1	2
*Gene flow prediction based on residence*	*2*	*2*	*1*
Foraging bout duration	1	1	NA
Tripping rate in 2012	3	2	1
*Gene flow prediction for tripping rate*	*1*	*2*	*3*
Directionality within foraging bouts	YES	YES	NO
Overall preference for directions	YES	NO	NO

Gene flow predictions are italicized and presented for net distance traveled, residence and tripping rate.

### Directionality of movement within foraging bouts

In both years we observed significant angular correlations for bumble bees (WS = 174.9, P < 0.0001 first year and WS = 152.0, P < 0.0001 second year) and honey bees (WS = 30.6, P = 0.04 first year and WS = 50.75, P = 0.001 second year) but not for leafcutting bees (WS = 5.95, P = 0.47 first year and WS = 13.78, P = 0.70 second year). These analyses were run on 818 directions over 79 foraging bouts in the first year and 746 directions over 70 foraging bouts in the second year for bumble bees; 248 directions over 41 foraging bouts in the first and 345 directions over 28 foraging bouts in the second year for honey bees; and finally, 125 directions over 37 foraging bouts in the first and 221 directions over 53 foraging bouts in the second year for leafcutting bees. Representative foraging bouts for each bee species, presented in S2 Fig, captured the presence or absence of directionality of movement within foraging bouts.

### Residence and foraging bout duration

Residence did not differ significantly between bumble bees and honey bees (F_1, 201_ = 1.38, P = 0.24). On average, bumble bees visited (mean ± se) 53.9 ± 8.1 flowers per foraging bout in a patch while honey bees visited 48.2 ± 9.5 flowers. Moreover, the duration of a foraging bout did not differ significantly between bumble bees and honey bees (235.6 ± 33.5 sec for bumble bees and 202.3 ± 33.0 sec for honey bees) (F_1, 176_ = 0.35, P = 0.56) (Table 1).

### Proportion of flowers tripped per raceme

**Fig 1:**
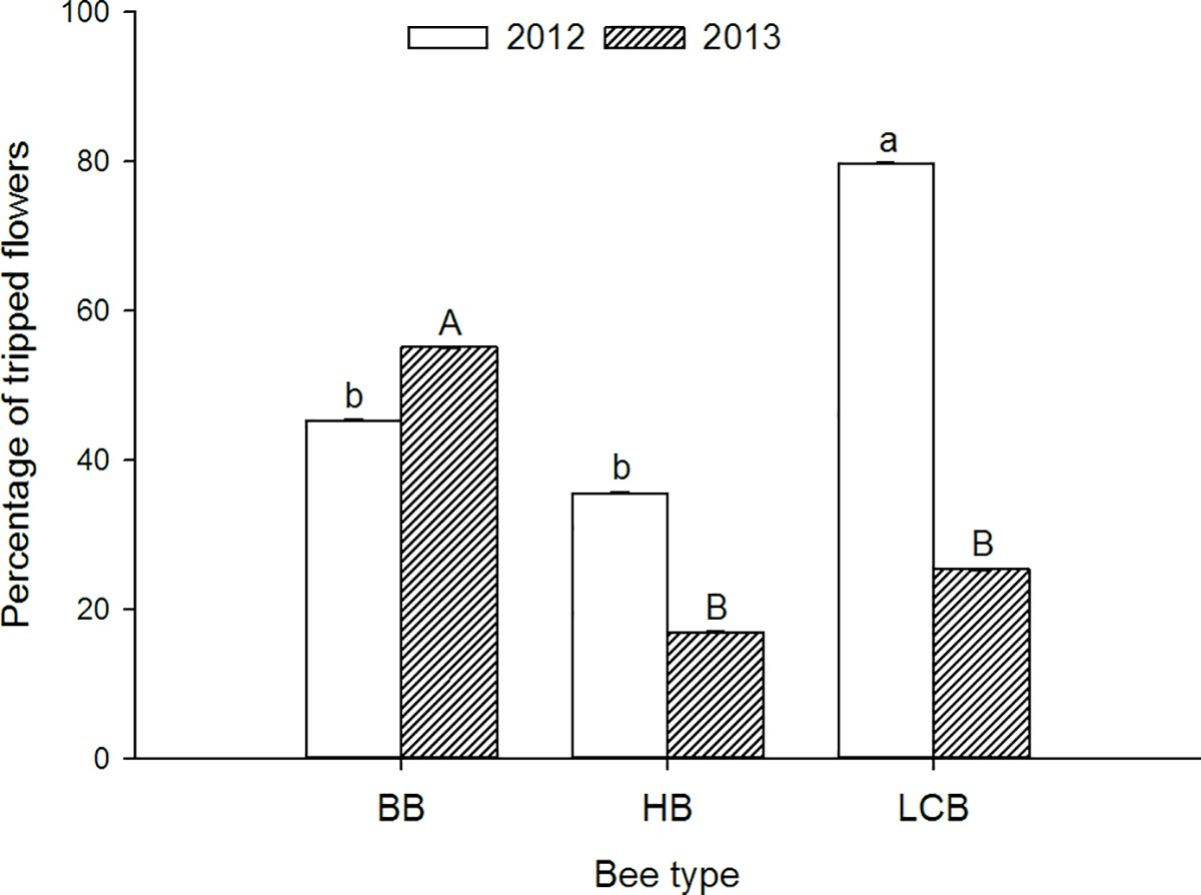
The impact of bee species and year on the tripping rate. The tripping rate is the proportion of flowers visited whose anthers and stigmas were released by a pollinator. Different letters indicate significant statistical differences among bee species with upper or lower case letters used for a given year.

Bee species (F_2,477_ = 11.1, P = 0.0001), year (F_1,477_ = 25.5, P = 0.0001) and the interaction between these two factors (F_2,477_ = 20.9, P = 0.0001) all affected the proportion of flowers tripped per raceme or tripping rate. Although bumble bees tripped the most flowers overall (mean ± se) (50.67% ± 0.03) relative to leafcutting bees (40.61% ± 0.04) and honey bees (22.69% ± 0.04), and although tripping rate was greater the first (52.06% ± 0.03) relative to the second year (37.85% ± 0.03), the tripping rate and relative ranking of the three bee species varied between years (Fig 1). Leafcutting bees were the best trippers the first year (LCB-BB: t_1, 477_ = 4.47, P < 0.0001 and LCB-HB: t_1, 477_ = 4.67, P < 0.001) while bumble bees and honey bees had similar tripping rates (BB-HB: t_1, 477_ = 1.71, P = 0.09). In contrast, the second year bumble bees tripped significantly more flowers relative to both honey bees (BB-HB: t_1, 477_ = 4.47, P < 0.001) and leafcutting bees (BB-LCB: t_1,477_ = 4.82, P < 0.001) which tripped a similar proportion of the flowers they visited (LCB-HB: t_1,477_ = 1.31, P = 0.19) (Fig 1). Only leafcutting bees tripped a significantly smaller proportion of visited flowers in the second relative to the first year (t_1, 477_ = 6.78, P < 0.0001); the proportion of tripped flowers did not differ between years for the other two bee species (BB: t_1, 477_ = 0.84, P = 0.40 and HB: t_1, 477_ = 1.80, P = 0.073).

### Overall preference for directions

**Fig 2:**
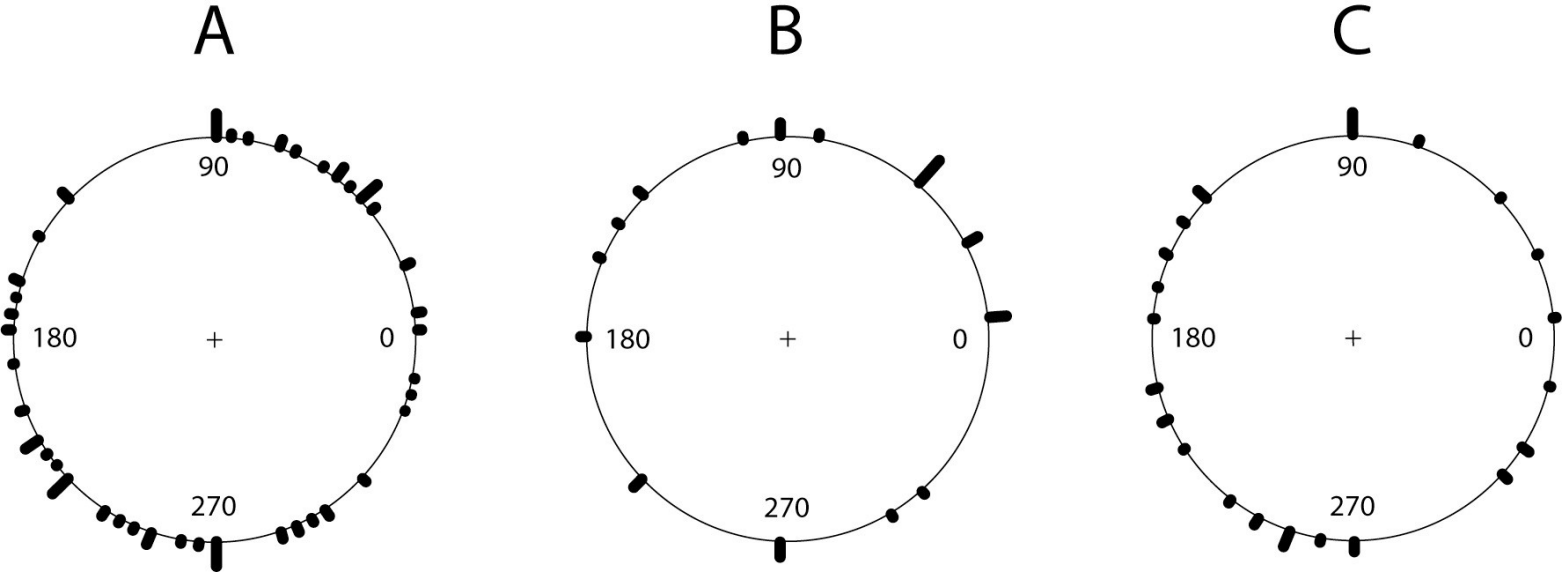
Overall directions flown by a) bumble bee, b) leafcutting bee and c) honey bee.

We detected no overall preferred directions for bumble bees in the first (W = 0.098, P > 0.10) or second year (W = 0.090, P > 0.10) (Fig 2A for year 1). The data sets consisted of 748 directions over 50 foraging bouts in the first and 662 directions over 42 foraging bouts in the second year. Similarly, leafcutting bees did not exhibit a preference for specific directions (W = 0.118, P > 0.10 in the first and W = 0.017, P > 0.10 in the second year) (Fig 2B for year 1) although the number of foraging bouts with five sequences or more was low both years for leafcutting bees (43 directions over 7 foraging bouts in the first and 114 directions over 13 foraging bouts in the second year). For honey bees, the uniformity test was statistically significant in both years, indicating that honey bees did not randomly select directions but preferred some directions (W = 0.418, P < 0.01 in the first and W = 0.29, P < 0.01 in the second year) (Fig 2C for year 1). Tests for honey bees used 187 directions over 20 foraging bouts in the first and 316 directions over 18 foraging bouts in the second year.

## Discussion

First, we discuss the distinct foraging behaviors observed for the three bee species. Second, each foraging behavior is ranked to predict the relative gene flow of the three bee species. Third, these predictions are tested against available gene flow data and the information is used to identify the foraging behaviors that most impact gene flow. Last, we discuss how knowledge of these behaviors can provide mechanisms to explain observed differences in gene flow amongst bee species and can help develop management practices to limit gene flow.

### Foraging behaviors of the three bee species

Because bumble bees and honey bees are both social bees and are larger than leafcutting bees, we expected their foraging behaviors to be more similar to each other than to the behavior of leacutting bees. As is illustrated below, this pattern holds true for some but not all foraging behaviors examined in this study.

#### Residence

Bumble bees and honey bees had similar average residence of approximately 50 flowers per patch. Noticeably, a previous study examining residence for a different bumble bee species foraging on oilseed rape obtained a value of 60 flowers for residence in 10 m in length patches [9]. This value is fairly similar to the 50+ flowers observed in the current study which used patches of 11m length with a different bumble bee species foraging on *M*. *sativa*. In the current study, foraging bout duration was similar between the two bee species, with bees spending, on average, a little over three minutes foraging in a patch. Although we could not directly quantify residence for leafcutting bees in the field, observations of their foraging behavior in a greenhouse setting indicated a lower residence for leafcutting bees relative to bumble bees. Leafcutting bees visited on average 12 flowers during a foraging bout relative to 45 flowers for bumble bees (J. Brunet unpublished work). These data indicate similar residence for honey bees and bumble bees and suggest both species have greater residence relative to leafcutting bees.

#### Directionality of movement

Both social bees exhibited directionality of movement within foraging bouts while the solitary bee species moved randomly among racemes. The current study used serial angular correlation tests to detect directionality of movement over multiple foraging bouts. Previous studies typically used Chi-square tests to detect departure from a random distribution for the number of times a pollinator moved in the same direction (angle 0) or changed direction by 45, 90, 135 or 180 degrees between pairs of consecutive flowers/racemes or plants and this test best applied to single foraging bouts [16, 28]. While directionality of movement was not detected for leafcutting bees using the serial angular correlation test in the current study, Collevatti et al. [28] reported directionality of movement for five solitary bees, *Augochlorella michaelis*, *Augochloropsis cupreola*, *Pseudocentron paulistana*, *Ceratinula* sp., *Melissodes sexcincta*, and two social bees, *Plebeia droryana*, *P*. *cf*. *nigriceps*, foraging on a tropical shrub weed *Triumfetta semitriloba*, using the chi-square tests. Future studies of other solitary and social bee species, using the serial angular correlation test, should help determine how common directionality of movement is in different bee species and whether the pattern observed between social and solitary bees in the current study is typical of bee species.

#### Distances traveled

Bumble bees and leafcutting bees traveled similar distances between consecutive racemes, and both species traveled greater distances than honey bees. However, all three bee species differed in net distances traveled, with bumble bees traveling greater net distances relative to honey bees, and both bumble bees and honey bees traveling longer net distances relative to leafcutting bees. Differences in net distances traveled among bee species could result from differences in behavior such as the presence of directionality of movement within foraging bouts for bumble bees and honey bees and its absence in leafcutting bees. Directionality of movement within foraging bouts tends to increase the net distance traveled by a bee [16]. However, the number of clips visited per foraging bout also varied among bee species and was smallest for leafcutting bees and largest for honey bees. The differences in the number of clips visited per bee species could reflect distinct behaviors or variation in observers’ ability to follow these bee species. Honey bees’ foraging bouts had the most clips but honey bees are not easier to follow in the field relative to bumble bees. Moreover, as mentioned earlier, leafcutting bees visited fewer flowers per foraging bout relative to bumble bees in a greenhouse setting where bees could be followed more easily. We therefore conclude that the differences in number of clips observed among bee species reflect differences in behavior among these species. Honey bees traveled shorter net distances and shorter distances between racemes relative to bumble bees. Moreover, leafcutting bees visited fewer racemes per foraging bout and lacked directionality of movement, behaviors which both tend to shorten net distances traveled. As expected, the distribution of net distances traveled was leptokurtic for each bee species, a pattern similar to the one observed for distances traveled between plants by one bumble bee species foraging on *Lotus corniculatus* [29].

#### Tripping rate

Leafcutting bees tripped the most flowers (80% tripping rate) the first year while bumble bees were the greatest trippers the second year (55%). Tripping rate was only significantly different between years for leafcutting bees with 80% tripping in the first and 25% in the second year. Leafcutting bees are known to be good trippers of *M*. *sativa* unless temperatures are cool [20, 30–31] and the second summer was cool in Wisconsin. Honey bees tend to have low tripping rates except at very high temperatures [32, 20]. A tripping rate of approximately 50% was also observed for bumble bees in a separate study [23]. Therefore, under temperatures typical of alfalfa seed-production fields, leafcutting bees are expected to be the best trippers, followed by bumble bees and finally, honey bees.

#### Overall preference for directions

Honeybees exhibited a preference for some directions while bumble bees and leafcutting bees were as likely to select any directions. To our knowledge, this is the first report of an overall preference for directions by bees. A statistical test to quantify the presence of overall preference for directions is also introduced in this study. A preference for certain directions in honey bees indicates that gene flow estimates measured from a given direction may not be representative of gene flow in other directions for this bee species. One potential explanation for such a pattern is that honey bees are influenced by the hive location when selecting directions. Honey bees preferred western directions in our study and their hive was located to the west of the experimental fields. Future studies should examine whether honey bees typically have a preference for certain directions and whether such preferences are affected by hive location. Preference for some directions will result in stronger gene flow in specific directions. To date, a pattern of non-uniform gene flow in different directions had only been reported in some wind-pollinated plants, where prevailing winds can affect the probability of pollen dispersal and gene flow [17].

#### Overall trends

The social bees most resembled each other and differed from the solitary bee species with respect to directionality of movement within a foraging bout and residence. Bumble bees most resembled leafcutting bees and differed from honey bees with respect to distances traveled between consecutive racemes and overall preference for a direction. All three bee species differed from each other in tripping rate and net distances traveled during a foraging bout. Future studies comparing the foraging behaviors of distinct bee species and of bee species foraging on different plant species will help determine the generality of the behavioral differences observed among bee species in this study.

### Foraging behaviors that most impact gene flow

#### Gene flow predictions based on behavior

Gene flow predictions based on the relative ranking of a behavior among bee species varied with specific behaviors (summarized in Table 1). Net distances traveled predicted greatest gene flow for bumble bees, followed by honey bees and finally leafcutting bees. Shorter dispersal distances are expected with higher residence [8], and, therefore, based on residence, similar gene flow was predicted for bumble bees and honey bees and lower gene flow for these two bee species relative to leafcutting bees. For tripping rate, we used the first year data to predict gene flow because it better reflected tripping rates under the temperatures in seed-production fields in the Pacific Northwest where leafcutting bees are the dominant pollinators. Based on tripping rate, we predicted honey bees to have the greatest gene flow, followed by bumble bees, and least leafcutting bees.

#### Testing gene flow predictions based on behavior

Previous studies that examined the distance traveled by the Roundup Ready (RR) gene transferred by pollinators via pollen in alfalfa seed-production fields indicated that honey bees have the greater probability of moving the RR gene long distance relative to leafcutting bees [21, 33–36]. There was a quick decline in the probability of finding any RR gene after 1,000 feet for leafcutting bees and closer to 3,000 feet for honey bees. Bumble bees are not used in alfalfa seed-production and there currently exist no gene flow data on this bee species foraging on *M*. *sativa* flowers. Both net distances traveled and tripping rate predicted lower gene flow for leafcutting bees relative to honey bees, and this ranking was supported by the empirical gene flow data. Residence, on the other hand, predicted higher gene flow for leafcutting bees relative to honey bees, a pattern not supported by the gene flow data.

#### Pollinator behavior and gene flow

The data presented here support the conclusions previously reached by Levey et al. [13–14] studying birds, that distances and directions traveled at a local scale could predict gene flow patterns at the landscape level. In the current study, honey bees traveled longer net distances within patches relative to leafcutting bees and these differences were maintained in the gene flow data. Unlike previous studies, however, where residence was inversely proportional to gene flow [8–9], residence did not predict gene flow patterns among bee species. Leafcutting bees had both the shortest residence and shortest gene flow distances. While these studies examined the impact of residence on gene flow for a given bee species, we examined its predictive power when comparing bee species. Within a bee species, features of the agricultural landscape including patch size, isolation distance and plant density are predicted to affect residence and subsequent gene flow [9, 23]. Factors that increase residence will tend to limit gene flow because visiting more flowers in a field increases the chance that the source pollen, for e.g. RR pollen, gets depleted within that field and is not moved to the subsequent field. When distinct bee species are compared, we suspect the pollen deposition curve or the pattern of pollen deposition over successive flowers visited may supersede residence on its impact on gene flow [12]. Future studies should elucidate this question. Finally, because pollen grains are not deposited on stigmas or removed from the anthers of untripped flowers, lower tripping rates will tend to increase the distance traveled by pollen [12]. We propose that tripping rate may, in fact, represent the strongest predictor of gene flow risk for distinct bee species. A pollinator that trips many flowers will increase seed set and may also limit gene flow risk relative to other pollinators with lower tripping rates. Many species in the family Fabaceae have a tripping mechanism and the impact of tripping rate on gene flow should be determined in other plant species. Moreover, for species without a tripping mechanism, our results beget the question whether pollinators that are inefficient at depositing pollen on a plant’s stigmas may be disproportionately responsible for long distance pollen dispersal events.

### Management practices to limit gene flow

Linking foraging behavior to gene flow can guide the design of management practices to limit gene flow. In *Medicago sativa*, leafcutting bees have the highest tripping rates and move genes shorter distances relative to honey bees [32, 20, 21, 35, 34]. Environmental factors can influence tripping rate; high temperatures increase the tripping rate of a bee and low temperatures can significantly decrease the tripping rate of leafcutting bees [32, 20, 30–31]. It is therefore recommended to grow *M*. *sativa* for seeds in warmer areas where these bees can survive well, tripping is high which can limit gene flow. Moreover, it is recommended to use honey bees in the warmest regions where high temperatures can increase their tripping rate which should decrease the distance traveled by pollen. Selection for increased ease of tripping of *M*. *sativa* cultivars should be pursued as a mean to help limit gene flow risk [37–39]. Although many of these practices are already adopted in alfalfa seed-production fields, the information provided here provides a mechanistic explanation by linking environmental factors, their impact on a specific behavior and predicted gene flow risk.

## Conclusions

All behaviors examined in this study differed among bee species. Both social bee species exhibited directionality of movement and had similar residence which differed from the random movement and lower residence of the solitary bee species. Tripping rate and net distance traveled differed among the three bee species. Bumble bees most resembled leafcutting bees and differed from honey bees with respect to distances traveled between consecutive racemes and overall preference for a direction. Tripping rate and net distances traveled, but not residence, predicted differences in gene flow among species. The use of bee species with high tripping rates and crops grown under environmental conditions known to increase tripping could reduce gene flow. This study illustrates how differences in foraging behaviors among bee species can affect gene flow and how linking foraging behaviors to gene flow can foster the development of management strategies to reduce gene flow. Although developed in one system, the approach developed here can be generalized to different plant/pollinator systems.

## Supporting information

S1 Fig Frequency distribution of net distances traveled by the three bee species. (TIF)

S2 Fig Representative foraging paths for a) bumble bee, b) honey bee, and c) leafcutting bee.
(TIF)
