# Supplementary figures and images for: Linking the foraging behavior of three bee species to pollen dispersal and gene flow
Source: PLoS One. 2019 Feb 26;14(2):e0212561. doi: 10.1371/journal.pone.0212561 (PMC6391023; doi:10.1371/journal.pone.0212561)

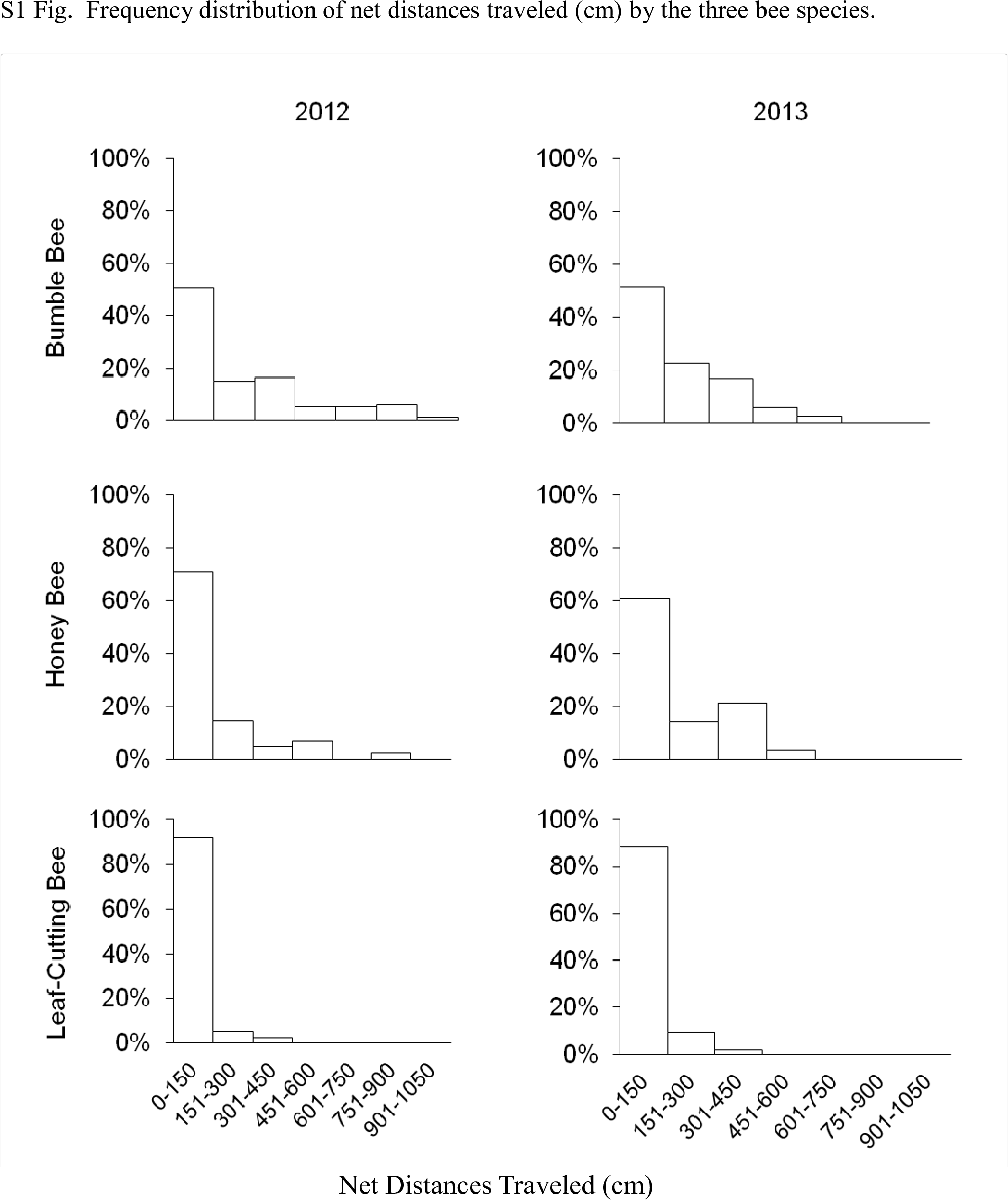

Supplement: S1 Fig — (TIF) [file pone.0212561.s001.tif]

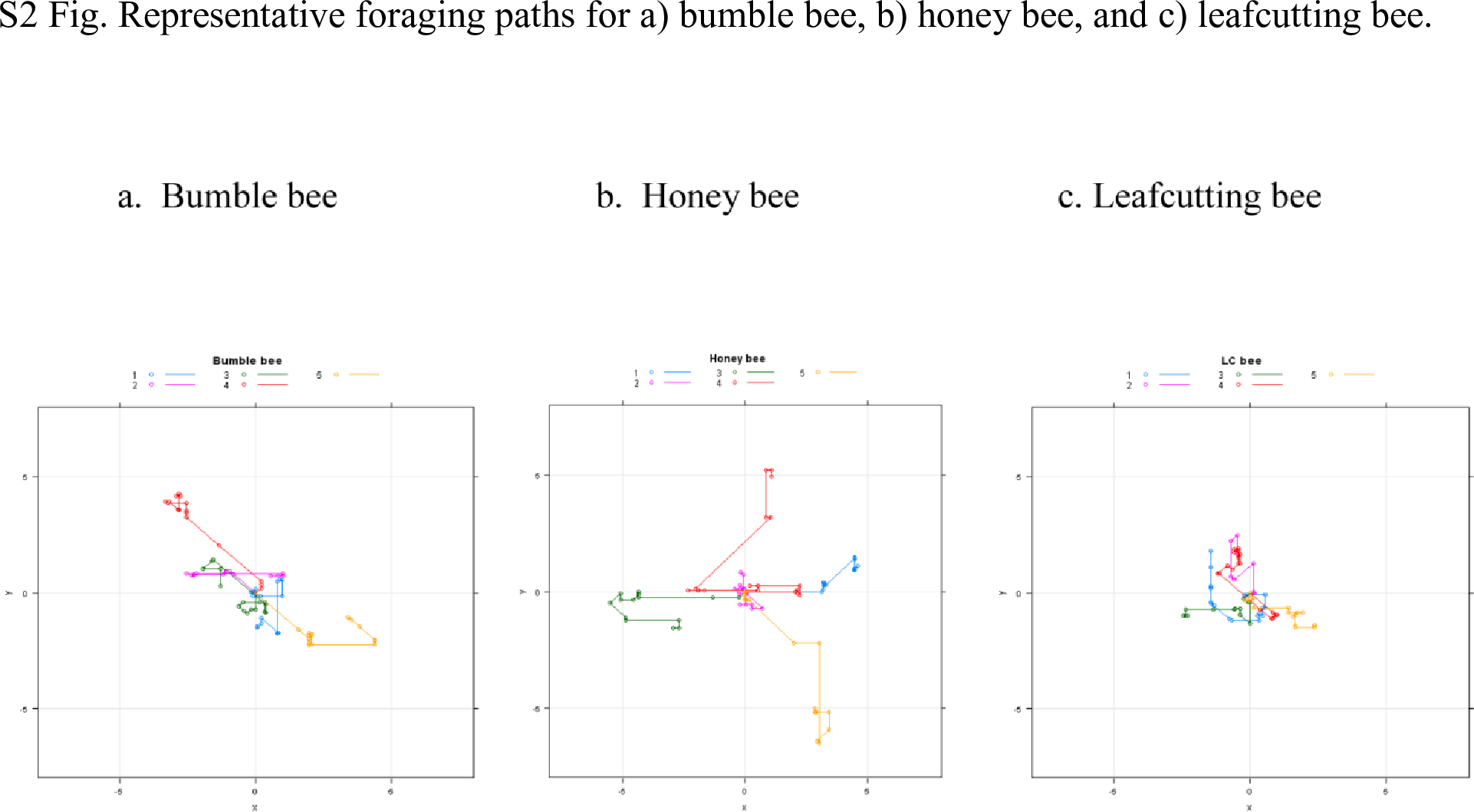

Supplement: S2 Fig — Representative foraging paths for a) bumble bee, b) honey bee, and c) leafcutting bee. (TIF) [file pone.0212561.s002.tif]
